# The influence of a high fat diet on bone and soft tissue formation in Matrix Gla Protein knockout mice

**DOI:** 10.1038/s41598-018-21650-0

**Published:** 2018-02-26

**Authors:** S. A. Lanham, F. R. Cagampang, R. O. C. Oreffo

**Affiliations:** 1 Bone and Joint Research Group, Centre for Human Development, Stem Cells and Regeneration, Human Development and Health, Institute of Developmental Sciences, Faculty of Medicine, University of Southampton, Southampton, SO16 6YD UK; 2 Maternal, Pregnancy, and Child Research Group, Human Development and Health, Institute of Developmental Sciences, Faculty of Medicine, University of Southampton, Southampton, UK

## Abstract

Studies suggest bone growth and development are influenced by maternal nutrition, during intrauterine and early postnatal life. This study assessed the role of MGP and a maternal high fat diet on vitamin K-dependent proteins’ gene expression and their impact on bone formation. Knockout (KO) offspring were smaller than wild type (WT) littermates, yet possessed the same volume of intrascapular brown adipose tissue. The total proportion of body fat was reduced, but only in animals on a control diet. Lung air volume was observed to be comparable in both KO and WT animals on the same diet. The degree of aortic calcification was reduced in KO animals maintained on a HF diet. KO females on the high fat diet showed reduced cortical bone volume and thickness in the femur and tibia. Gene expression levels of GGCX and VKOR were reduced in control fed KO animals suggesting a potential link between gene expression levels of MGP, GGCX, and VKOR and total volumes of bone, calcified soft tissue, and iBAT; with implications for modulation of body length and mass. Our results confirm the important role for vitamin K in bone and calcified soft tissue, but now extend this role to include iBAT.

## Introduction

Epidemiological and animal studies indicate that environmental factors, such as maternal nutrition, influence disease risk in later life^1–4^. Indeed, numerous studies have shown that intrauterine growth restriction, a proxy measure of poor prenatal environment, can affect cardiovascular and metabolic control in animals and humans in a nutritionally abundant postnatal environment^5–8^. Furthermore, dysfunction of the vascular system can lead to a number of diseases including hypertension, atherogenesis, type 2 diabetes, coronary heart disease, metabolic syndrome, and obesity. We and others have shown that bone development and osteoporosis can be added to this growing list of non-communicable diseases affected by intrauterine environment^9^. However, the mechanisms of action of the maternal diet on offspring bone development are currently unknown.

Previously, we have shown that a maternal high fat diet combined with an offspring high fat diet resulted in alterations in offspring bone structure at six weeks of age^10^, and, crucially, that matrix gla protein (MGP) gene expression levels were negatively correlated with bone structural parameters. In addition, a post-weaning high-fat diet reduced the serum levels of uncarboxylated MGP. These data show that MGP is altered by both the maternal and offspring high-fat diet and indicate, potentially, an important role for MGP in bone development.

To better understand the interaction of MGP, maternal high fat diet and offspring bone development; we used a mouse MGP knockout model, and assessed hard tissue (bone phenotype), soft tissue and body composition (as fat levels may alter the potential for bone formation), and vasculature calcification (which is regulated by MGP). We demonstrate the importance of MGP and the vitamin K metabolism enzymes VKOR and GGCX in the development of bone and adipose tissues and implications therein for development within a rich dietary environment.

## Results

### Body Size

Upon comparison to animals on the same diet, MGP knockout (KO) animals were found to be lighter than wild type (WT) littermates. In addition, control (C)-fed animals were also shorter than controls (Table 1). The MGP KO mice maintained on a high fat (HF) diet had the same mass and body length as C-fed WT animals (Table 1). No differences were seen between the sexes.

**Table 1:** Body Composition by sex from MGP mice.

Sex	Males				Females			
Genotype	WT		KO		WT		KO	
	Mean (SD)		Mean (SD)		Mean (SD)		Mean (SD)	
Diet	Control	High Fat	Control	High Fat	Control	High Fat	Control	High Fat
	(n = 8)	(n = 14)	(n = 8)	(n = 7)	(n = 11)	(n = 11)	(n = 9)	(n = 6)
Mass/g	11 (3)^ab^	14 (3)^b^	8 (2)^a^	10 (2)^a^	11 (2)^a^	14 (3)^b^	7 (2)^c^	11 (2)^a^
Body Length/mm	46 (3)^a^	51 (3)^b^	40 (3)^c^	47 (3)^ab^	45 (3)^a^	51 (4)^b^	39 (3)^c^	46 (3)^ab^
Fat Vol/mm^3^	1100 (300)^a^	2000 (500)^b^	500 (200)^c^	1200 (200)^a^	1200 (400)^a^	2200 (500)^b^	400 (200)^c^	1600 (600)^a^
Soft Tissue Vol/mm^3^	6100 (1300)^ab^	8000 (2000)^b^	3900 (1000)^a^	5800 (1200)^a^	5700 (1000)^a^	7600 (1600)^b^	3100 (500)^c^	5600 (900)^a^
Bone Vol/mm^3^	260 (50)^a^	340 (70)^b^	220 (30)^a^	290 (50)^ab^	260 (40)^a^	360 (70)^b^	210 (30)^a^	280 (50)^a^
Calcified Tissue Vol/mm^3^	0^a^	0^a^	8 (1)^b^	9 (1)^b^	0^a^	0^a^	8 (1)^b^	8 (2)^b^
Fat as % of body	15 (2)^a^	19 (3)^b^	11 (2)^c^	16 (3)^ab^	16 (2)^a^	21 (2)^b^	11 (3)^c^	20 (3)^b^
Soft Tissue as % of body	81 (2)^ac^	77 (3)^b^	84 (1)^c^	80 (3)^ab^	80 (2)^a^	75 (2)^b^	83 (3)^a^	76 (3)^b^
Bone as % of body	3.6 (0.2)^a^	3.3 (0.2)^a^	4.8 (0.9)^b^	3.9 (0.4)^a^	3.7 (0.3)^a^	3.6 (0.2)^a^	5.6 (1.1)^b^	3.5 (0.5)^a^
Calcified Tissue as % of body	0^a^	0^a^	0.19 (0.05)^b^	0.13 (0.02)^b^	0^a^	0^a^	0.23 (0.05)^b^	0.11 (0.03)^c^

For each parameter measured, values with different superscript letters are significantly different from each other (p < 0.05) within the same sex.

### Body composition

**Figure 1:**
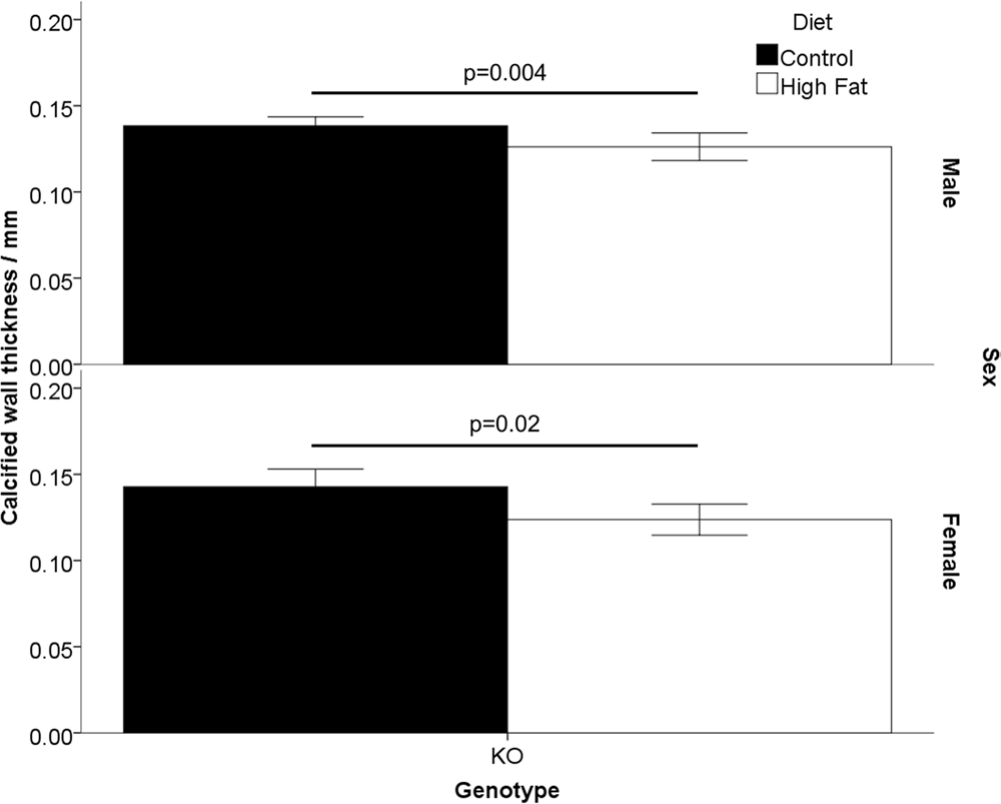
Calcified Wall Thickness of Abdominal Aorta of MGP Genotypes at 25 days of Age. MGP heterozygous males and females were mated. At the start of pregnancy dams were fed either a control or a high-fat diet. These diets were continued throughout pregnancy and after birth. Wild type (WT) and MGP knockout (KO) offspring were analysed at 25 days of age. The thickness of calcification of the abdominal aorta wall was determined by CT. The section of the aorta from the diaphragm to the split for the femoral arteries was analysed. Bars show calcified wall thickness for each MGP genotype and diet. Graphs show by sex, n = 4–6 per group. Graphs show mean plus 95% confidence limits.

Female HF-fed animals displayed a greater fat and tissue volume than C-fed animals with the same genotype, whereas HF-fed males only showed greater fat volume (Table 1). For both sexes, HF-fed WT animals showed a greater bone volume than C-fed WT mice, whereas bone volume was not altered between KO animals (Table 1). Only MGP KO animals contained calcified soft tissues; the volume was the same regardless of the diet (See supplementary material). Hence, diet composition did not appear to influence the degree of tissue calcification caused by the MGP KO genotype. However, the HF-fed KO mice had a 0.4 fold higher mass and 0.2 fold longer body length than C-fed KO animals (Table 1). When the wall thickness of the abdominal aorta was analysed, the C-fed KO animals had a significantly thicker calcified wall than KO animals fed the HF diet (Fig. 1).

Full details of the body composition of the offspring are presented in Table 1. For both sexes, as a percentage of total body volume, HF-fed animals had a 0.2–0.6 fold higher proportion of fat and a lower proportion of soft tissue compared to C-fed animals with the same genotype. Interestingly, the proportion of bone was conserved within HF-fed (WT and KO), and C-fed WT animals. Control fed KO mice had around 0.4 fold more bone as a proportion of body volume (Table 1).

### Intrascapular Brown Adipose Tissue (iBAT)

**Figure 2:**
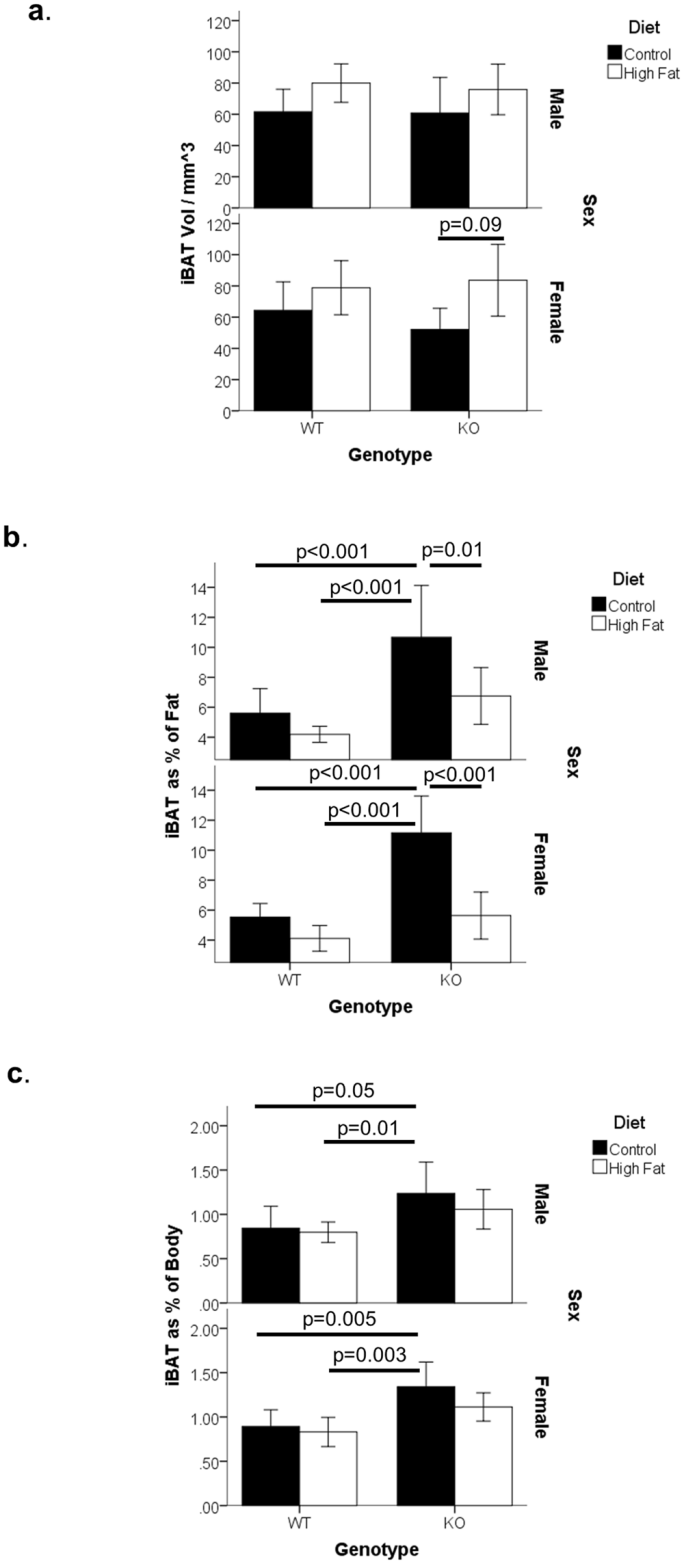
Intrascapular Brown Adipose Tissue (iBAT) of MGP Genotypes at 25 days of Age. MGP heterozygous males and females were mated. At the start of pregnancy dams were fed either a control or a high-fat diet. These diets were continued throughout pregnancy and after birth. Wild type (WT) and MGP knockout (KO) offspring were analysed at 25 days of age. Graphs shows (**a**) Intrascapular brown adipose tissue (iBAT) volume as determined by CT. (**b**) BAT volume as a percentage of total fat volume. (**c**) iBAT volume as a percentage of total body volume. For all graphs, results are shown by sex, n = 6–14 per group. Bar charts show mean plus 95% confidence limits.

Animals maintained on a control diet displayed similar volumes of iBAT, regardless of the MGP genotype (Fig. 2a). Similarly, animals fed the HF diet had comparable volumes of iBAT, regardless of the MGP genotype. The volume of iBAT tended to be higher in the HF-fed group within the same MGP genotype, although these did not reach statistical significance.

For iBAT volume as a percentage of the total fat volume, C-fed MGP KO mice of both sexes displayed a significantly higher percentage (over 2 fold) than WT animals on either diet (Fig. 2b). For iBAT volume as a percentage of the total body volume, C-fed MGP KO mice contained a significantly higher percentage (approx. 0.5 fold) than WT mice fed either diet (Fig. 2c).

When iBAT volume was normalised to body size (statistics were based on the residuals following multiple regression against mass and body length) and analysed by genotype, MGP KO animals were found to have a significantly increased iBAT volume (p = 0.036). This suggests MGP KO mice had a higher proportion of iBAT for their size and thus MGP may influence iBAT volume.

### Lung structure

**Figure 3:**
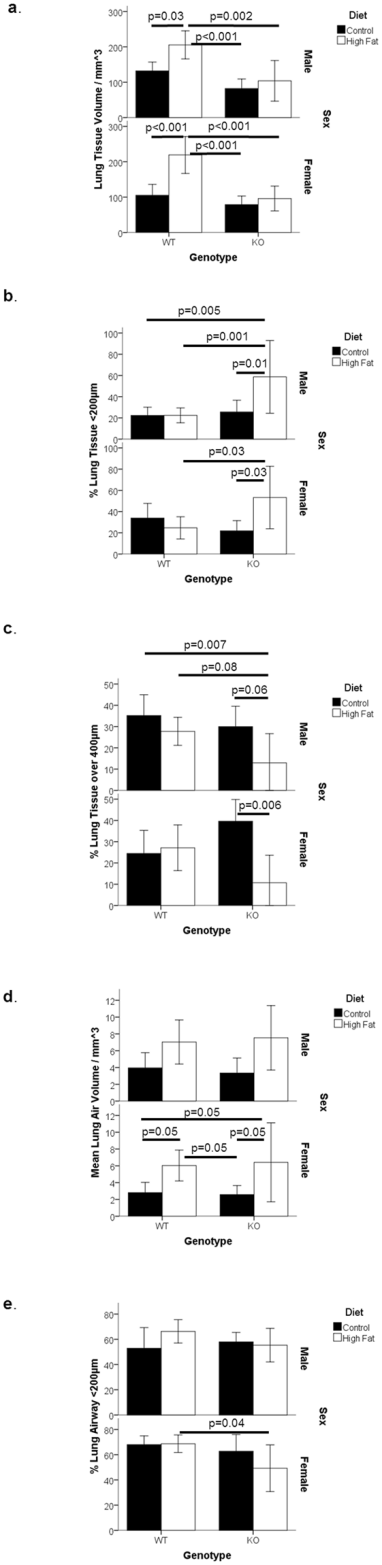
Lung Structure of MGP Genotypes at 25 days of Age. MGP heterozygous males and females were mated. At the start of pregnancy dams were fed either a control or a high-fat diet. These diets were continued throughout pregnancy and after birth. Wild type (WT) and MGP knockout (KO) offspring were analysed at 25 days of age. Graphs shows (**a**). Total lung tissue volume as determined by CT. (**b**) Percentage of total lung tissue volume with thickness of less than 200 µm. (**c**) Percentage of total lung tissue volume with thickness greater than 400 µm. (**d**) Total lung air volume. (**e**) Percentage of total lung airway volume with thickness of less than 200 µm. For all graphs, results are shown by sex, n = 6–14 per group. Bar charts show mean plus 95% confidence limits.

For each sex, lung tissue volume was significantly higher (approx. 0.6–2 fold) in WT mice maintained on a high fat diet in comparison to any of the other groups (Fig. 3a and supplementary material). Figure 3b shows the percentage of the lung tissue which was 200 µm in width or lower upon ultrastructure examination by CT. For both male and female cohorts, the HF-fed KO animals displayed a higher proportion (approx.1.2 fold) of these smaller lung tissue structures than the other groups. These differences corresponded with a significant reduction (approx. 1.6 fold) in the percentage of lung tissue over 400 µm in size in this group, particularly in males (Fig. 3c). These differences indicated a combination of the HF diet and MGP KO genotype altered the size of the tissue composition of the lungs.

In contrast, female mice maintained on a HF diet showed a significantly higher lung air volume (approx. 1 fold) than animals fed the C diet, within the same genotype and across the different genotypes (Fig. 3d and supplementary material). A similar trend was found in male mice, but was not statistically significant. Whilst HF-fed mice demonstrated the same lung air volume, the thickness of the airways demonstrated a different distribution. Thus, KO females displayed a lower proportion of airways less than 200 µm in thickness (Fig. 3e). Lung air volume was normalised to body size (statistics are based on the residuals following multiple regression against mass and body length). When analysed by diet, no significant differences were found (p = 0.09). However, when assessed by genotype, the KO animals were found to have a larger lung air volume than expected. Hence, MGP may influence lung air volume.

### Femur structure

Compared to C-fed WT animals, HF-fed WT animals showed significantly different results in femoral structure for most parameters studied (Table 2), particularly in females. In contrast, HF-fed KO animals showed few significantly different results when compared to C-fed KO mice (Table 2). In addition, C-fed KO mice showed few differences compared to C-fed WT animals (particularly in males), yet most parameters when compared to HF-fed WT mice (Table 2). The differences in the results observed may have been due to differences in animal mass between the groups (Table 1).

**Table 2:** Bone Structural Parameters from MGP mice by sex.

Sex	Males				Females			
Genotype	WT		KO		WT		KO	
	Mean (SD)		Mean (SD)		Mean (SD)		Mean (SD)	
Diet	Control	High Fat	Control	High Fat	Control	High Fat	Control	High Fat
	(n = 8)	(n = 14)	(n = 8)	(n = 7)	(n = 11)	(n = 11)	(n = 9)	(n = 6)
Femur								
Length/mm	10.7 (0.6)^ab^	11.5 (0.7)^b^	9.7 (0.5)^c^	10.6 (0.7)^ac^	10.5 (0.6)^a^	11.5 (0.9)^b^	9.5 (0.6)^c^	10.6 (0.6)^ab^
BV/mm^3^	5.6 (1.8)^ab^	8.2 (3.1)^b^	4.4 (1.2)^a^	5.0 (1.6)^a^	5.6 (1.6)^a^	9.1 (3.4)^b^	3.8 (1.3)^a^	5.1 (2.4)^a^
BvTv	24 (13)^a^	40 (14)^b^	9 (6)^a^	15 (6)^a^	18 (11)^a^	38 (14)^b^	6 (3)^a^	21 (12)^a^
BsBv	34 (22)^ab^	22 (5)^b^	51 (23)^a^	42 (23)^ab^	42 (26)^ab^	22 (6)^b^	57 (32)^a^	28 (5)^ab^
TbTh/mm	0.15 (0.05)^ab^	0.22 (0.07)^b^	0.11 (0.03)^a^	0.13 (0.05)^a^	0.14 (0.05)^a^	0.21 (0.05)^b^	0.10 (0.03)^a^	0.16 (0.02)^ab^
TbSp/mm	0.60 (0.20)^a^	0.45 (0.18)^b^	0.70 (0.07)^a^	0.63 (0.12)^ab^	0.65 (0.18)^a^	0.46 (0.16)^b^	0.73 (0.08)^a^	0.62 (0.19)^ab^
SMI	2.3 (0.4)^a^	2.2 (0.6)^b^	2.9 (0.6)^a^	2.5 (0.5)^ab^	2.6 (0.4)	2.1 (0.7)	2.7 (0.4)	2.6 (0.4)
TbPF/mm^−1^	9 (4)^ab^	5 (4)^b^	20 (7)^c^	13 (5)^ac^	15 (11)^a^	6 (6)^b^	21 (3)^a^	11 (6)^ab^
ConnD/mm^−3^	71 (22)	82 (19)	94 (65)	91 (55)	98 (64)	77 (12)	61 (63)	69 (24)
Cortical BV/mm^3^	0.9 (0.3)^a^	1.4 (0.5)^b^	0.6 (0.1)^a^	0.8 (0.2)^a^	0.9 (0.1)^a 12^	1.5 (0.4)^b 1^	0.5 (0.1)^c 1^	0.8 (0.2)^ac 2 ˅^
Cortical Th/mm	0.13 (0.02)^a^	0.17 (0.04)^b^	0.11 (0.01)^a^	0.12 (0.02)^a^	0.14 (0.01)^a 1^	0.18 (0.04)^b 1^	0.10 (0.01)^c 1^	0.12 (0.02)^ac 2˅^
MidD/mm	1.11 (0.07)^ac^	1.19 (0.05)^b^	1.07 (0.07)^c^	1.17 (0.05)^ab^	1.09 (0.06)^a^	1.21 (0.08)^b^	1.06 (0.06)^a^	1.13 (0.05)^ab^
MidWT/mm	0.13 (0.02)^ab^	0.14 (0.02)^b^	0.11 (0.01)^a^	0.12 (0.01)^a^	0.13 (0.01)^ab 1^	0.15 (0.02)^b 12^	0.11 (0.01)^c 12^	0.12 (0.02)^ac 2˅^
Tibia								
Length/mm	11.1 (0.6)^ab^	11.7 (0.6)^a^	10.6 (0.3)^b^	10.9 (0.8)^ab^	11.2 (0.3)^ab^	11.7 (0.7)^a^	10.3 (0.6)^b^	10.9 (0.4)^ab^
BV/mm^3^	5.7 (0.8)^a^	8.3 (1.0)^b^	4.1 (0.5)^c^	4.9 (1.1)^ac^	5.6 (0.6)^a 12^	8.7 (1.2)^b 1^	3.7 (0.9)^c 12^	4.7 (0.6)^ac 2˅^
BvTv	24 (6)	25 (5)	22 (3)	26 (9)	21 (2)	29 (9)	22 (7)	23 (6)
BsBv	30 (4)	33 (5)	31 (3)	31 (6)	32 (3)	31 (4)	32 (7)	30 (3)
TbTh/mm	0.15 (0.02)	0.15 (0.02)	0.15 (0.01)	0.15 (0.02)	0.15 (0.02)	0.16 (0.02)	0.14 (0.03)	0.15 (0.02)
TbSp/mm	0.50 (0.08)	0.39 (0.07)	0.51 (0.04)	0.43 (0.07)	0.48 (0.04)^a 12^	0.36 (0.07)^b 1^	0.49 (0.05)^a 1^	0.53 (0.07)^a 2^^
SMI	2.6 (0.2)	2.9 (0.1)	2.6 (0.2)	2.5 (0.4)	2.9 (0.4)^a^	2.7 (0.4)^ab^	2.3 (0.1)^b^	2.6 (0.3)^ab^
TbPF/mm^−1^	11 (2)	13 (3)	11 (1)	11 (5)	13 (2)	11 (4)	10 (2)	11 (3)
ConnD/mm^-3^	70 (9)^a^	138 (53)^b^	77 (10)^a^	96 (12)^ab^	80 (22)	113 (19)	77 (15)	87 (9)
Cortical BV/mm^3^	1.0 (0.1)^a^	1.4 (0.1)^b^	0.7 (0.1)^c^	0.8 (0.2)^ac^	1.0 (0.1)^a 1^	1.5 (0.2)^b 1^	0.6 (0.1)^c 1^	0.8 (0.1)^c 2˅^
Cortical Th/mm	0.15 (0.02)^a^	0.19 (0.01)^b^	0.12 (0.01)^c^	0.13 (0.02)^ac^	0.15 (0.01)^a 1^	0.20 (0.01)^b 1^	0.11 (0.01)^c 12^	0.13 (0.01)^c 2˅^
Vertebra								
Length/mm	2.1 (0.2)^ab^	2.3 (0.2)^a^	1.8 (0.1)^b^	2.2 (0.3)^a^	2.1 (0.2)^a^	2.4 (0.3)^b^	1.8 (0.1)^c^	2.2 (0.2)^ab^
BV/mm^3^	4.1 (1.0)^ab^	5.3 (1.5)^a^	3.1 (0.6)^b^	3.9 (0.8)^ab^	3.9 (0.9)^a 1^	5.8 (1.8)^b 1^	2.8 (0.6)^a 2^^	4.1 (1.1)^ab 12^
BvTv	53 (6)	51 (16)	53 (9)	46 (10)	48 (8)	48 (9)	51 (10)	42 (15)
BsBv	29 (3)	28 (7)	30 (4)	33 (6)	31 (3)	29 (5)	30 (5)	34 (8)
TbTh/mm	0.13 (0.01)	0.14 (0.03)	0.13 (0.01)	0.12 (0.02)	0.12 (0.01)	0.14 (0.02)	0.13 (0.02)	0.12 (0.03)
TbSp/mm	0.16 (0.01)	0.17 (0.03)	0.16 (0.02)	0.17 (0.01)	0.17 (0.02)	0.18 (0.02)	0.17 (0.02)	0.18 (0.02)
SMI	1.2 (0.3)	1.1 (0.6)	1.2 (0.3)	1.4 (0.5)	1.4 (0.3)	1.3 (0.4)	1.4 (0.5)	1.5 (0.7)
TbPF/mm^−1^	3 (2)	3 (4)	3 (2)	5 (4)	5 (2)	4 (3)	5 (3)	6 (6)
ConnD/mm^−3^	89 (19)	70 (19)	90 (17)	93 (20)	83 (17)	71 (18)	88 (31)	81 (25)

Abbreviations are BV: bone volume, BvTv: trabecular bone volume to total volume, BsBv: Bone surface to bone volume ratio, TbTh: Trabecular thickness, TbSp: Trabecular spacing, SMI: Structural model index, TbPF: Trabecular pattern factor, ConnD: connectivity density, Cortical Th: cortical thicknesss, MidD: Midshaft diameter, MidWT: Midshaft cortical wall thickness. For each parameter measured, values with different superscript letters are significantly different from each other (p < 0.05) within the same sex. For each parameter measured, values with different superscript numbers are significantly different from each other for body size–adjusted data (p < 0.05) within the same sex.

Table 2 shows the femoral structural parameters normalised to body size (statistics are based on the residuals following multiple regression against mass and body length). HF-fed KO females showed a lower than expected cortical bone volume, cortical thickness, and midshaft wall thickness (Table 2).

### Tibia structure

The tibia was assessed by CT; the results are shown in Table 2. The HF-fed WT animals showed significantly different results in tibial structure for bone volume, trabecular spacing (females), cortical bone volume, and cortical wall thickness when compared to C-fed WT animals. In contrast, HF-fed MGP KO animals showed no differences when compared to C-fed KO mice (Table 2). In addition, C-fed KO mice showed significantly different results for bone volume, cortical bone volume, and cortical wall thickness when compared to C-fed and HF-fed WT animals (Table 2). However, the HF-fed KO mice did not differ to C-fed WT mice for any of the parameters assessed, except for cortical bone volume and cortical wall thickness in females (Table 2). To determine if these differences were due to differences in animal mass between the groups (Table 1), the tibial structural parameters were normalised to body size. As seen with the femur, HF-fed MGP KO females showed a lower cortical bone volume and cortical thickness than expected for their size (Table 2).

### Vertebra structure

Analysis of the 3^rd^ lumbar vertebrae showed HF-fed WT females had significant differences in length and bone volume when compared to C-fed WT animals (Table 2). HF-fed KO animals showed no differences in comparison to C-fed WT or HF-fed WT animals. C-fed KO female mice only differed in length to the other groups. When the results were normalised to body size (Table 2), C-fed KO females were found to have a higher total bone volume.

### Femur qPCR

Femoral expression levels for the genes assessed did not differ between C- and HF-fed WT animals (Table 3). *Periostin* expression was significantly reduced (0.4–0.7 fold) in KO animals compared to diet controls in both sexes (except HF-fed females). As expected, *Mgp* expression was found to be 10–25 fold lower in the KO animals compared to WT (Table 3). Although levels were expected to be zero, a valid qPCR CT value of around 35 was detected in these samples (c.f. CT value of 26 for *Beta*-actin). Both *Ggcx* and *Vkor* displayed a lower level of expression in female KO mice; however, this was only statistically significant in the C-fed KO animals compared to HF-fed WT females (0.5 fold for *Ggcx*) and C-fed WT mice (0.5 fold for *Vkor*). Expression levels of *Bmp-2* were lower (0.5 fold) in HF-fed KO males compared to C-fed KO males. Estradiol Receptor alpha (*ERα*) levels were reduced 0.5 fold in female C-fed KO mice compared to female C-fed WT animals.

**Table 3:** Mean Normalised Gene Expression Levels in Femur.

Sex	Males				Females			
Genotype	WT		KO		WT		KO	
	Mean (SD)		Mean (SD)		Mean (SD)		Mean (SD)	
Diet	Control	High Fat	Control	High Fat	Control	High Fat	Control	High Fat
	(n = 8)	(n = 14)	(n = 8)	(n = 7)	(n = 11)	(n = 11)	(n = 9)	(n = 6)
*Osteocalcin*	0.20 (0.11)	0.25 (0.07)	0.25 (0.19)	0.08 (0.01)	0.19 (0.08)	0.19 (0.09)	0.14 (0.11)	0.08 (0.02)
*Gas6*	0.03 (0.02)	0.04 (0.03)	0.06 (0.02)	0.04 (0.01)	0.04 (0.02)	0.04 (0.01)	0.06 (0.03)	0.03 (0.02)
*Periostin*	2.4 (1.1)^ab^	2.4 (1.1)^b^	0.7 (0.4)^c^	1.1 (0.4)^ac^	2.9 (0.9)^a^	2.4 (1.1)^ac^	0.7 (0.3)^bc^	1.5 (1.1)^c^
*Mgp*	0.078 (0.044)^a^	0.061 (0.030)^a^	0.004 (0.003)^b^	0.007 (0.003)^b^	0.093 (0.053)^a^	0.055 (0.025)^ab^	0.004 (0.003)^b^	0.003 (0.002)^b^
*Ggcx*	0.0014 (0.0007)	0.0013 (0.0005)	0.0010 (0.0004)	0.0010 (0.0003)	0.0012 (0.0006)^ab^	0.0013 (0.0004)^a^	0.0006 (0.0004)^b^	0.0010 (0.0002)^ab^
*Vkor*	0.022 (0.010)	0.026 (0.012)	0.014 (0.007)	0.014 (0.003)	0.026 (0.012)^a^	0.019 (0.007)^ab^	0.012 (0.004)^b^	0.014 (0.005)^ab^
*Bmp-1*	0.7 (0.4)	0.8 (0.3)	0.6 (0.3)	0.6 (0.4)	0.7 (0.3)	0.6 (0.2)	0.4 (0.1)	0.6 (0.3)
*Bmp-2*	0.004 (0.001)^ab^	0.005 (0.003)^ab^	0.007 (0.002)^a^	0.003 (0.003)^b^	0.005 (0.003)	0.004 (0.003)	0.003 (0.002)	0.005 (0.003)
*Bmp-4*	0.05 (0.02)	0.06 (0.03)	0.08 (0.03)	0.06 (0.03)	0.06 (0.04)	0.05 (0.02)	0.08 (0.05)	0.03 (0.02)
*Leptin*	0.019 (0.014)	0.023 (0.020)	0.020 (0.013)	0.021 (0.009(	0.015 (0.007)	0.024 (0.011)	0.023 (0.018)	0.026 (0.026)
*Leptin Receptor*	0.33 (0.04)	0.36 (0.28)	0.57 (0.23)	0.46 (0.20)	0.53 (0.32)	0.33 (0.16)	0.49 (0.28)	0.35 (0.12)
*Sclerostin*	0.0013 (0.0004)	0.0025 (0.0011)	0.0021 (0.0010)	0.0021 (0.0008)	0.0023 (0.0012)	0.0019 (0.0008)	0.0021 (0.0015)	0.0023 (0.0013)
*Estradiol*	ND	ND	ND	ND	ND	ND	ND	ND
*Estradiol Receptor* α	0.13 (0.05)	0.13 (0.04)	0.10 (0.04)	0.12 (0.05)	0.16 (0.07)^a^	0.13 (0.05)^ab^	0.06 (0.03)^b^	0.09 (0.03)^ab^
*Estradiol Receptor* β	ND	ND	ND	ND	ND	ND	ND	ND
*ACE*	0.31 (0.07)	0.38 (0.24)	0.22 (0.08)	0.34 (0.10)	0.32 (0.09)	0.29 (0.12)	0.19 (0.09)	0.22 (0.09)
*Angiotensinogen*	0.16 (0.04)	0.26 (0.17)	0.33 (0.17)	0.31 (0.18)	0.29 (0.18)	0.19 (0.09)	0.32 (0.17)	0.21 (0.04)
*Vegf*_120_	0.05 (0.02)	0.07 (0.02)	0.08 (0.03)	0.10 (0.07)	0.08 (0.08)	0.08 (0.03)	0.08 (0.05)	0.05 (0.03)
*Vegf*_164_	0.08 (0.03)	0.12 (0.06)	0.08 (0.02)	0.11 (0.05)	0.11 (0.04)	0.13 (0.05)	0.07 (0.03)	0.08 (0.04)
*Vegf*_188_	0.02 (0.01)	0.02 (0.02)	0.02 (0.01)	0.01 (0.010)	0.04 (0.04)	0.02 (0.01)	0.01 (0.01)	0.01 (0.00)
*Vegf*_*total*_	0.11 (0.03)	0.15 (0.06)	0.12 (0.04)	0.10 (0.02)	0.16 (0.07)	0.15 (0.05)	0.10 (0.04)	0.10 (0.02)
*Gapdh*	14 (11)	7 (5)	12 (8)	12 (9)	10 (7)	9 (3)	11 (5)	9 (6)

For each parameter measured, values with different superscript letters are significantly different from each other within the same sex (p < 0.05). ND – not detected.

### Aorta qPCR

Although found at very low levels (valid qPCR CT values of around 36 were detected, c.f. CT value of 22 for *Beta*-actin), *Osteocalcin* expression was lower in C-fed KO males compared to C-fed WT males (Table 4). *Mgp* expression was found to be around 20 fold lower in the KO animals compared to controls (Table 4). Although levels were expected to be zero, a valid qPCR CT value of ~32 was detected in these samples. *Ggcx* levels were reduced (0.5 fold) in C-fed KO animals compared to C-fed WT mice. *Bmp-4* levels were lower (0.5 fold) in KO animals compared to controls, but this was only statistically significant in males (Table 4). *Leptin* levels were higher (approx. 2 fold) in KO animals compared to controls, but this was only statistically significant in males (Table 4). *Sclerostin* levels were reduced 0.6 fold in KO animals, particularly in males. *ERα* levels were reduced 0.5 fold in C-fed KO animals, particularly in females (Table 4). *Angiotensinogen* levels were raised 2.5 fold in C-fed KO animals. *Vegf*_120_ levels were increased 2 fold in female C-fed KO females compared to C-fed WT females, and *Vegf*_188_ level were reduced 0.5 fold in male C-fed KO compared to male C-fed WT animals (Table 4). Glyceraldehyde-3-Phosphate Dehydrogenase (*Gapdh*) levels were reduced 3–10 fold in KO animals compared to WT mice (Table 4).

**Table 4:** Mean Normalised Gene Expression Levels in Aorta.

*Sex*	*Males*				*Females*			
*Genotype*	*WT*		*KO*		*WT*		*KO*	
	*Mean (SD)*		*Mean (SD)*		*Mean (SD)*		*Mean (SD)*	
*Diet*	*Control*	*High Fat*	*Control*	*High Fat*	*Control*	*High Fat*	*Control*	*High Fat*
	*(n* = *8)*	*(n* = *14)*	*(n* = *8)*	*(n* = *7)*	*(n* = *11)*	*(n* = *11)*	*(n* = *9)*	*(n* = *6)*
*Osteocalcin*	0.00005 (0.00001)^a^	0.00004 (0.00001)^ab^	0.00002 (0.00001)^b^	0.00002 (0.00001)^ab^	0.00004 (0.00004)	0.00002 (0.00001)	0.00004 (0.00006)	0.00006 (0.00003)
*Gas6*	0.009 (0.003)	0.014 (0.005)	0.014 (0.006)	0.013 (0.006)	0.010 (0.003)^a^	0.014 (0.007)^ab^	0.013 (0.005)^ab^	0.018 (0.006)^b^
*Periostin*	0.10 (0.02)	0.09 (0.03)	0.08 (0.05)	0.09 (0.04)	0.08 (0.02)	0.09 (0.04)	0.07 (0.03)	0.10 (0.04)
*Mgp*	0.013 (0.004)^a^	0.016 (0.008)^a^	0.001 (0.000)^b^	0.001 (0.001)^b^	0.017 (0.009)^a^	0.016 (0.008)^a^	0.001 (0.001)^b^	0.001 (0.001)^b^
*Ggcx*	0.0006 (0.0003)^a^	0.0005 (0.0002)^ab^	0.0003 (0.0001)^b^	0.0004 (0.0001)^ab^	0.0006 (0.0002)^a^	0.0005 (0.0002)^a^	0.0002 (0.0001)^b^	0.0004 (0.0002)^ab^
*Vkor*	0.005 (0.002)	0.005 (0.001)	0.003 (0.001)	0.003 (0.001)	0.005 (0.002)	0.005 (0.002)	0.003 (0.001)	0.004 (0.001)
*Bmp-1*	0.011 (0.004)^ab^	0.013 (0.006)^a^	0.007 (0.003)^b^	0.007 (0.002)^ab^	0.009 (0.001)^ab^	0.012 (0.007)^a^	0.006 (0.003)^b^	0.009 (0.001)^ab^
*Bmp-2*	0.0001 (0.0000)	0.0002 (0.0001)	0.0001 (0.0000)	0.0001 (0.0001)	0.0001 (0.0001)	0.0001 (0.0001)	0.0001 (0.0001)	0.0001 (0.0001)
*Bmp-4*	0.014 (0.007)^a^	0.014 (0.002)^a^	0.005 (0.002)^b^	0.004 (0.002)^b^	0.013 (0.005)^a^	0.011 (0.005)^ab^	0.007 (0.004)^ab^	0.005 (0.003)^b^
*Leptin*	0.003 (0.002)^a^	0.006 (0.004)^ab^	0.010 (0.005)^bc^	0.014 (0.003)^c^	0.005 (0.004)	0.007 (0.006)	0.008 (0.003)	0.010 (0.008)
*Leptin Receptor*	0.002 (0.001)	0.003 (0.002)	0.002 (0.001)	0.005 (0.006)	0.002 (0.001)	0.003 (0.001)	0.003 (0.002)	0.006 (0.007)
*Sclerostin*	0.011 (0.005)^a^	0.017 (0.004)^b^	0.002 (0.002)^c^	0.003 (0.002)^c^	0.009 (0.005)^ab^	0.011 (0.005)^b^	0.003 (0.002)^c^	0.004 (0.005)^ac^
*Estradiol*	ND	ND	ND	ND	ND	ND	ND	ND
*Estradiol Receptor α*	0.008 (0.003)^a^	0.007 (0.003)^ab^	0.004 (0.002)^b^	0.007 (0.002)^ab^	0.008 (0.002)^ab^	0.010 (0.003)^b^	0.004 (0.002)^c^	0.006 (0.003)^ac^
*Estradiol Receptor β*	ND	ND	ND	ND	ND	ND	ND	ND
*ACE*	0.02 (0.01)	0.03 (0.01)	0.03 (0.02)	0.03 (0.01)	0.02 (0.01)^a^	0.03 (0.02)^a^	0.04 (0.01)^b^	0.02 (0.01)^a^
*Angiotensinogen*	0.002 (0.003)^a^	0.001 (0.000)^a^	0.008 (0.005)^b^	0.007 (0.004)^ab^	0.004 (0.003)^a^	0.001 (0.000)^a^	0.010 (0.003)^b^	0.004 (0.002)^a^
*Vegf*_120_	0.005 (0.005)^a^	0.011 (0.006)^ab^	0.012 (0.004)^ab^	0.017 (0.003)^b^	0.007 (0.004)^a^	0.010 (0.003)^ab^	0.014 (0.004)^b^	0.012 (0.004)^ab^
*Vegf*_164_	0.022 (0.010)	0.028 (0.014)	0.024 (0.014)	0.038 (0.004)	0.016 (0.005)	0.028 (0.008)	0.025 (0.012)	0.023 (0.012)
*Vegf*_188_	0.048 (0.017)^a^	0.038 (0.021)^ab^	0.019 (0.007)^b^	0.048 (0.012)^a^	0.028 (0.010)	0.033 (0.011)	0.024 (0.017)	0.040 (0.020)
*Vegf*_*total*_	0.08 (0.03)	0.08 (0.04)	0.06 (0.03)	0.11 (0.02)	0.06 (0.02)	0.07 (0.02)	0.07 (0.03)	0.08 (0.03)
*Gapdh*	2.5 (1.0)^a^	1.4 (0.7)^a^	0.3 (0.2)^b^	0.6 (0.6)^b^	1.7 (0.5)^a^	1.3 (0.5)^a^	0.3 (0.2)^b^	0.6 (0.2)^b^

For each parameter measured, values with different superscript letters are significantly different from each other within the same sex (p < 0.05). ND – not detected.

### Correlation of Gene Expression to Body Composition

Five genes from the femur (*Ggcx*, *Vkor*, *Mgp*, *Gas6*, and *Periostin*) and 7 from the aorta (*Sclerostin, Bmp-1, ER alpha, Angiotensinogen, Ggcx*, *Vkor*, and *Mgp)* showed a significant correlation to bone volume as a percentage of total body volume (Table 5). Of these 12 genes, only femoral expression of *Gas6* and aortic expression of *Angiotensinogen* showed a positive correlation, with the remainder displaying a negative correlation with bone volume. Indeed, both these genes showed the opposite correlation to all other genes for the parameters assessed, shown in Table 5.

**Table 5:** Linear regression analysis between gene expression levels and body parameters.

Body Parameter	Aortic *Sclerostin*				Aortic *Bmp-1*				Aortic *ER alpha*				Aortic *Angiotensinogen*			
	β	r^2^	F	p	β	r^2^	F	p	β	r^2^	F	p	β	r^2^	F	p
% Bone	−0.4	0.18	12.5	0.001	−0.4	0.19	12.9	0.001	−0.5	0.27	20.6	<0.001	0.7	0.49	50.1	<0.001
% Fat	0.4	0.17	12.2	0.001	0.4	0.17	11.2	0.001	0.5	0.26	19.5	<0.001	−0.6	0.34	26.9	<0.001
% Soft tissue	−0.4	0.13	8.8	0.004	−0.4	0.13	8.2	0.006	−0.4	0.20	13.7	<0.001	0.5	0.24	17.1	<0.001
% Brown Adipose Tissue	−0.5	0.23	16.1	<0.001	−0.5	0.27	20.0	<0.001	−0.4	0.18	11.0	0.002	0.4	0.17	10.1	0.003
% Calcified soft tissue	−0.7	0.42	42.5	<0.001	−0.5	0.24	17.6	<0.001	−0.6	0.41	37.8	<0.001	0.7	0.55	64.7	<0.001
% Lung tissue	0.2	0.04	2.3	NS	0.0	0.00	0.0	NS	−0.1	0.01	0.6	NS	−0.1	0.01	0.8	NS
% Lung airways	0.2	0.00	0.0	NS	−0.2	0.04	1.5	NS	−0.1	0.00	0.1	NS	−0.1	0.02	0.6	NS
Length	0.5	0.25	19.2	<0.001	0.5	0.21	15.4	<0.001	0.5	0.27	20.4	<0.001	−0.6	0.35	27.9	<0.001
Mass	0.5	0.30	24.4	<0.001	0.4	0.15	9.8	0.003	0.4	0.17	11.0	0.002	−0.6	0.34	26.9	<0.001
Body Parameter	**Aortic** ***Ggcx***				**Aortic** ***Vkor***				**Aortic** ***Mgp***				Femoral *Gas6*			
	β	r^2^	F	p	β	r^2^	F	p	β	r^2^	F	p	β	r^2^	F	p
% Bone	−1.3	0.33	28.2	<0.001	−1.1	0.24	17.5	<0.001	−0.6	0.24	18.0	<0.001	0.7	0.27	20.3	<0.001
% Fat	4.2	0.12	8.0	0.006	4.8	0.16	10.7	0.002	2.4	0.11	7.1	0.01	−2.4	0.11	6.6	0.01
% Soft tissue	−2.8	0.07	4.0	0.05	−3.6	0.12	7.4	0.009	−1.6	0.06	3.9	0.05	1.7	0.07	3.9	0.05
% Brown Adipose Tissue	−0.4	0.23	15.8	<0.001	−0.3	0.11	6.2	0.02	−0.3	0.25	17.8	<0.001	0.1	0.03	1.9	NS
% Calcified soft tissue	−0.2	0.40	37.4	<0.001	−0.1	0.26	20.1	<0.001	−0.1	0.55	70.4	<0.001	0.06	0.15	9.6	0.003
% Lung tissue	−0.4	0.04	2.5	NS	−0.08	0.00	0.1	NS	−0.05	0.00	0.1	NS	0.2	0.02	1.2	NS
% Lung airways	−0.03	0.02	0.7	NS	−0.04	0.03	1.4	NS	−0.00	0.00	0.01	NS	0.02	0.01	0.5	NS
Length	8.1	0.33	28.3	<0.001	7.5	0.30	23.4	<0.001	3.8	0.21	14.7	<0.001	−2.4	0.08	4.5	0.04
Mass	5.2	0.31	25.6	<0.001	4.7	0.28	21.3	<0.001	2.8	0.26	20.1	<0.001	−1.3	0.05	2.8	NS
**Body Parameter**	**Femoral** *Ggcx*				**Femoral** ***Vkor***				Femoral *Mgp*				**Femoral** ***Periostin***			
	β	r^2^	F	p	β	r^2^	F	p	β	r^2^	F	p	β	r^2^	F	p
% Bone	−0.9	0.19	12.4	0.001	−0.8	0.17	11.6	0.001	−0.7	0.20	13.4	0.001	−1.1	0.38	30.7	<0.001
% Fat	3.0	0.07	4.2	0.05	2.1	0.04	2.4	NS	1.4	0.03	1.9	NS	3.8	0.14	8.2	0.006
% Soft tissue	−2.0	0.04	2.3	NS	−1.2	0.02	1.0	NS	−0.6	0.01	0.4	NS	−2.5	0.08	4.1	0.05
% Brown Adipose Tissue	−0.4	0.19	11.7	0.001	−0.2	0.08	4.3	0.04	−0.3	0.21	14.1	<0.001	−0.4	0.23	14.4	<0.001
% Calcified soft tissue	−0.1	0.24	16.7	<0.001	−0.1	0.25	19.1	<0.001	−0.1	0.47	49.6	<0.001	−0.2	0.52	53.6	<0.001
% Lung tissue	−0.1	0.00	0.2	NS	−0.06	0.00	0.07	NS	−0.1	0.01	0.4	NS	−0.3	0.03	1.4	NS
% Lung airways	0.03	0.02	0.6	NS	−0.01	0.00	0.04	NS	−0.03	0.13	6.1	0.02	−0.02	0.05	1.8	NS
Length	5.4	0.17	11.1	0.002	3.9	0.10	6.1	0.02	2.7	0.08	5.1	0.03	7.2	0.36	27.7	<0.001
Mass	3.2	0.14	8.9	0.004	3.1	0.15	9.9	0.003	2.3	0.15	9.4	0.003	5.4	0.45	40.2	<0.001

% values are as percentage of total body volume. NS: Not statistically significant.

As summarised in Table 5, the 12 genes assessed also showed a significant correlation to calcified tissue volume as a percentage of total body volume. As expected, *Mgp* expression showed the highest degree of negative correlation (femoral and aortic), although rather surprisingly, femoral *Periostin* and aortic *Angiotensinogen* expression displayed a level of correlation similar to that of *Mgp* expression.

All the genes examined, with the exception of femoral *Gas6*, showed a significant negative correlation to percentage of iBAT; the highest r^2^ values were observed with *Ggcx* (femoral and aortic), *Mgp* (femoral and aortic), femoral *Periostin*, and aortic *Sclerostin* and *Bmp-1* (Table 5).

Aortic expression of *Sclersotin*, *Ggcx*, *Vkor*, and *Mgp* together with femoral *Periostin* expression showed highly significant positive correlations with body length and mass, whereas aortic *Angiotensinogen* expression showed significant negative correlations (Table 5).

There was negligible correlation observed between any of the genes analysed and tissue volume; either total body tissue or lung tissue (Table 5).

The relevance of the femoral expression of the 5 genes and bone volume was confirmed by multivariant analysis (Table 6). In addition, the relevance of aortic expression of the 7 genes in body length and mass are shown in Table 6. Femoral and aortic expression of the selected genes was relevant in the volume of calcified soft tissue, total fat volume, and iBAT. There was no evidence of a significant correlation between femoral and aortic gene expression with body soft tissue volume or lung tissue volume (Table 6).

**Table 6:** Linear regression analysis between gene expression levels and body parameters.

Multivariant analysis	Body Parameter	r	r^2^	F	p
	% Bone	0.86	0.75	23.6	<0.001
	% Fat	0.59	0.34	4.2	0.004
Femoral VKDP Genes:	% Soft tissue	0.48	0.23	2.5	0.05
*Gas6*	% Brown Adipose Tissue	0.64	0.41	5.2	0.001
*Periostin*	% Calcified soft tissue	0.89	0.79	29.2	<0.001
*Mgp*	% Lung Tissue	0.40	0.16	1.5	NS
*Ggcx*	% Lung airways	0.63	0.40	3.6	0.02
*Vkor*	Length	0.67	0.45	6.6	<0.001
	Mass	0.68	0.46	6.7	<0.001
	% Bone	0.61	0.37	10.5	<0.001
Aortic VKDP Genes:	% Fat	0.44	0.19	4.3	0.009
*Mgp*	% Soft tissue	0.36	0.13	2.7	0.05
*Ggcx*	% Brown Adipose Tissue	0.53	0.28	6.7	0.001
*Vkor*	% Calcified soft tissue	0.78	0.60	27.1	<0.001
	% Lung Tissue	0.26	0.07	1.3	NS
	% Lung airways	0.20	0.04	0.5	NS
	Length	0.62	0.38	11.0	<0.001
	Mass	0.62	0.39	11.3	<0.001
	% Bone	0.76	0.57	16.7	<0.001
Aortic Non-VKDP Genes:	% Fat	0.73	0.53	14.3	<0.001
*Sclerostin*	% Soft tissue	0.65	0.43	9.4	<0.001
*Bmp-1*	% Brown Adipose Tissue	0.61	0.37	6.8	<0.001
*ER alpha*	% Calcified soft tissue	0.85	0.72	32.1	<0.001
*Angiotensinogen*	% Lung Tissue	0.27	0.07	0.9	NS
	% Lung airways	0.33	0.11	1.1	NS
	Length	0.71	0.51	13.0	<0.001
	Mass	0.69	0.47	11.1	<0.001
Femoral VKDP and Aortic VKDP Genes	% Bone	0.88	0.78	15.5	<0.001
combined	% Fat	0.67	0.45	3.5	0.004
	% Soft tissue	0.58	0.34	2.3	0.05
	% Brown Adipose Tissue	0.72	0.52	4.5	0.001
	% Calcified soft tissue	0.92	0.85	25.0	<0.001
	% Lung Tissue	0.50	0.25	1.4	NS
	% Lung airways	0.64	0.41	2.0	0.09
	Length	0.78	0.60	6.6	<0.001
	Mass	0.77	0.59	6.3	<0.001
Femoral and all Aortic Genes	% Bone	0.93	0.87	15.9	<0.001
combined	% Fat	0.82	0.67	4.8	<0.001
	% Soft tissue	0.77	0.59	3.4	0.004
	% Brown Adipose Tissue	0.82	0.67	4.4	0.001
	% Calcified soft tissue	0.95	0.90	21.1	<0.001
	% Lung Tissue	0.66	0.43	1.8	NS
	% Lung airways	0.84	0.70	3.1	0.02
	Length	0.82	0.68	4.9	<0.001
	Mass	0.83	0.68	5.0	<0.001

% values are as percentage of total body volume. NS: Not statistically significant.

## Discussion

We have previously shown that MGP expression levels together with a maternal and offspring HF diet altered bone development. This study assessed the effect of MGP knockout offspring, with a maternal HF diet, on bone phenotype and body composition of the offspring, due to the close inter-relationship between bone and fat, and the regulation of vascular calcification by MGP. Interestingly, over-expression of MGP is lethal soon after birth^11^ due to a lack of branching of the lung vasculature.

We assessed the gene expression, in bone and aorta, of a number of proteins dependent on vitamin K, bone development, and vascular function. These were the vitamin K-dependent proteins (Osteocalcin, Gas6, Periostin, and Mgp), enzymes involved in the vitamin K cycle (Ggcx and Vkor), proteins involved in bone growth and metabolism (Bmp-1, Bmp-2, Bmp-4, Leptin, Leptin Receptor, Sclerostin, Estradiol, Estradiol Receptor α, and Estradiol Receptor β), proteins involved in the renin–angiotensin system (Angiotensin Converting Enzyme and Angiotensinogen), and proteins involved in angiogenesis (Vegf120, Vegf164, and Vegf188).

It is interesting that one of the commonly used housekeeping genes for qPCR analysis (*Gapdh*) was reduced in expression in MGP KO animals fed either diet. One possible explanation is reduced GAPDH production from the increased requirements of NADPH^12^ due to the increased oxidative stress of the HF diet^13^, and the process of calcification inducing stress^14^ and apoptosis in the aorta of the MGP KO animals. Gene expression levels of clotting factors were not assessed as hepatic vitamin K levels were not expected to reduce during the 3 week study period, as both diets contained the same amount of vitamin K.

The role of MGP in lung development has been studied by the Boström^11,15–17^ and Rannels^18,19^ groups. Their data showed; (i) MGP is expressed in the lung near the epithelial tips of distal buds^19^, (ii) over-expression of *Mgp* inhibits vascular growth in the lung by limiting BMP-4 activity and *Vegf* expression^11,15^, (iii) MGP KO mice exhibit excessive vascular branching^15^ and finally, (iv) MGP acts as an inhibitory morphogen, acting antagonistically to BMP-4 in an activator/inhibitor reaction-diffusion dynamic system in lung vascular development^11^. While Boström’s group studied lung from 4 week old KO animals, they did not discuss airway volume or lung tissue volumes. Interestingly, our diets appeared to alter lung air volume, given KO animals fed a HF diet has significantly higher air volume than WT animals fed the C diet, despite both groups of animals having the same mass, length and body composition. Interestingly lung tissue volume was the same in all groups except the WT HF group which had a larger volume. Hence, lung tissue volume may be proportional to size, or the abnormal lung vasculature reported by Boström^15^ in KO animals may prevent further tissue growth expected with the HF diet. An abnormal vasculature and accompanying reduced nutrient supply would explain the increased proportion of small volume tissue and reduced proportion of large volume tissue we found in the KO animals fed a high fat in the current study. However, the HF-fed KO animals displayed a lung air volume the same as HF-fed WT animals with a lung tissue volume half that of the WT animals. This was as a consequence of the KO animals having a higher proportion of wider airway than WT animals. Hence, our studies demonstrated diet and genotype alter lung phenotype.

While the total body fat volume appeared to alter with size across all groups, the volume of iBAT was the same in both WT and KO animals on the same diet, despite these animals being different in size, indicating MGP protein levels are potentially linked to iBAT volume, but not white adipose tissue (WAT) volume. Bone morphogenetic proteins (BMPs) are known to influence adipose tissue; BMP-4 promote the differentiation of WAT to beige adipose^20^, whereas BMP-7 directs iBAT adipogenesis^21^. In addition, MGP is known to bind BMP-2^22^ and BMP-4^23^ to prevent soft tissue calcification. It is not known if MGP binds BMP-7, although BMP-7 appears to prevent vascular calcification^24^. If MGP also bound BMP-7, then KO mice would be expected to produce more iBAT for their size. This occurs in C-fed KO animals, but not in HF-fed KO animals. Therefore, the HF-diet itself may be reducing BMP-4 and/or BMP-7 protein levels. However, the HF-diet did not alter gene expression levels of BMP-4. It is possible the increased proportion of iBAT seen in the C-fed KO mice is simply due to their smaller size and therefore an increased need for heat for thermoregulation (from iBAT), which reduces their overall fat volume (energy source).

The additional mass produced by the HF-fed KO animals made them indistinguishable from C-fed WT mice in body composition.

The total volume of calcified tissue was comparable in control and high fat fed KO animals. High fat fed KO animals were significantly larger than control fed KO mice. Hence, the HF-fed group showed an 11% reduction in calcified wall thickness compared to C-fed KO animals. Again, this demonstrates how diet and MGP genotype can altered the phenotype of the animals. It is not clear what mechanisms would reduce the level of calcified tissue on a HF diet, especially as this diet can be used to induce diabetes and subsequent vascular calcification. It is known that vascular calcification, in the absence of MGP, is due to uninhibited BMP protein levels^25^. Therefore, to reduce calcification, the diet must reduce BMP protein levels, or suppress other osteogenic pathways implicated in this calcification process^26,27^. It is possible the HF diet is inhibiting osteoblast activity^28^, thereby reducing the level of vascular calcification. This would also account for the reduced body proportion of bone seen in the HF-fed KO group compared to the C-fed KO animals.

Our data suggested KO animals fed a C diet had a higher proportion of bone for body size (particularly in the females), compared to the other groups. There was no increase in bone volume in the HF-fed KO females. The “lack of increase” expected due to the MGP KO genotype appears to be due to a reduction in cortical bone volume in HF-fed KO females, seen when normalised to body size. These results demonstrate the HF diet had altered the effect the MGP KO genotype had on growth and bone structure compared to the C diet. With altered cortical thickness, observed in our study, *sclerostin* levels from osteocytes might also be expected to be altered. However, this was not seen in the femur, but was seen in the aorta; with reduced *sclerostin* expression levels in C- and HF-fed KO mice. This is contrary to that seen in mouse aorta derived vascular smooth muscle cell (VSMC) calcification *in vitro*, where *Sclerostin* expression increased with the level of calcification^29^. Interestingly, the level of *Sclerostin* expression seen in the femur was comparable to that seen in the aorta of the KO animals, with the WT animals showing 3–5 × higher levels of expression in the aorta than the KO mice. Nevertheless, it is not clear whether serum sclerostin levels would be altered as a consequence of aorta or bone expression levels^30^. In addition, serum sclerostin levels do not necessarily correlate to bone quality^31^ and cortical and trabecular bone can demonstrate opposite expression levels in response to stimuli^32^. Hence, the effect of MGP on cortical bone requires further investigation.

Expression levels for the Vitamin K-dependent protein (VKDP) genes assessed did not differ between C- and HF-fed WT animals. This is in keeping with our previous data, where we showed no differences at 6 weeks of age, between C-fed WT offspring and WT offspring fed a HF diet for gestation and the first 3 weeks postnatally, before being switched to the C diet^10^. This latter group is more representative of the HF feeding schedule used here.

For C-fed KO animals, *Periostin*, *Vkor*, and *ER alpha* (in females) gene expression levels were reduced in the femur. In contrast, for HF fed KO animals, only *Periostin* gene expression was reduced in the femur. Thus, the reduction in gene expression of *Periostin* was independent of diet, whereas the other gene expression levels were diet specific. Similarly for the aorta, C-fed KO animals showed increased *Angiotensinogen* and *Vegf*120 gene expression, and reduced gene expression of *Ggcx*, *Bmp-4*, and *Gapdh*. HF-fed KO animals showed reduced gene expression of *Bmp-4* and *Gapdh*. Therefore the reduction in *Bmp-4* and *Gapdh* gene expression was independent of diet, whereas the other gene expression levels were diet specific. Therefore, specific VKDP gene expression was altered at specific sites by both MGP levels and the diet. In a model system of type 1 diabetes in rats, Doyon *et al*.^33^ studied the aorta and found increased calcification corresponded with reduced levels of carboxylated MGP (cMGP) and carboxylase (produced by GGCX), but no alteration in *Vkor* expression. Kaesler *et al*.^34^ used an adenine model of chronic kidney disease with aortic calcification in rats, and reported reduced GGCX activity and increased uncarboxylated MGP in the aorta. Using a similar model, McCabe *et al*.^35^ found reduced *Vkor* and *Ggcx*, but increased *Mgp* expression in the aorta. These studies are in agreement with our data and would indicate that in the calcifying aorta there are low levels of cMGP and reduced activity of GGCX. Carboxylation of MGP is required for MGP binding activity to BMPs. Clearly, a reduction in cMGP levels would increase aortic calcification. As stated above, the high fat diet may be altering the calcification process and thereby reducing the effects of the low cMGP levels.

Multivariant analysis of the expression levels of 3 vitamin K-relevant genes from the aorta (*Ggcx*, *Vkor*, and *Mgp*) showed significant correlations to body length and mass. Similar analysis for 5 genes from the femur (*Ggcx*, *Vkor*, *Mgp*, *Gas6*, and *Periostin*) showed a significant correlation to bone volume as a percentage of total body volume, as well as lung airway volume. Furthermore, multivariant analysis of these 8 genes combined showed significant correlations to the volume of calcified soft tissue, and iBAT.

We believe that the high fat diet caused sequestration of vitamin K within fat depots. The lack of availability of vitamin K to extra-hepatic tissues causes a reduction in the function of MGP, thereby affecting vascular calcification by not inhibiting BMPs, which in turn may result in altered iBAT. In addition, these low levels of vitamin K may alter GGCX and VKOR levels, resulting in altered vitamin K recycling.

Through gene expression analysis and Micro-CT analysis, we have confirmed, and extended, the ways in which MGP can alter lung structure. In addition, we suggest MGP expression and the HF diet alters bone formation in a sex specific manner. We also suggest a potential link between gene expression levels of MGP, GGCX, and VKOR and total volumes of bone, calcified soft tissue, and iBAT; with implications for modulation of body length and mass. Overall, our results confirm the important role for vitamin K in bone and calcified soft tissue, but now extend this role to include iBAT.

## Materials and Methods

### Experimental Design and Animal Care

All animal experimentation was performed and approved under license from the Home Office in accordance with the Animals (Scientific Procedures) Act (1986). All mice were raised within the University of Southampton Biomedical Research Facility and were housed in appropriate environments in rooms maintained at 22 ± 2 °C with a 12 h light:12 h dark cycle.

### MGP Knockout Mice

MGP+/− mice were generated by Karsenty and colleagues^36^, and obtained from Dr. Deepak Srivastava’s lab at the Gladstone Institute of Cardiovascular Disease (University of California, San Francisco) with the permission of Professor Gerard Karsenty. Heterozygous mice were bred to obtain homozygous MGP−/− mice, as well as MGP +/+ littermate control mice.

### High Fat Diet

At 13–18 weeks of age, female MGP+/− C57BL/6 strain mice were mated with aged-matched MGP+/− C57BL/6 males. Females were mated to separate males, and after confirmation of mating (presence of a vaginal plug) were individually housed and allocated either a control (C, n = 11 dams) diet of standard chow RM-1 (7.5% kcal fat, 17.5% kcal protein, 75% kcal carbohydrate; Special Diet Services, Witham, Essex, UK) or a high-fat diet (HF, n = 14 dams; 45% kcal fat, 20% kcal protein, 35% kcal carbohydrate; Special Diet Services diet 824053). These C or HF diets were continued throughout pregnancy and lactation until the offspring were harvested at 25 days of age. Animals were killed by cervical dislocation. Where possible for each dam, one wild type and one knockout (for both male and female) were included in the analysis. All dams produced litters containing knockout offspring.

### MGP Genotyping

Wild type MGP gene was detected using primers MGP-5 + : GCCACAATTTCTGCATCCT-GC and OMGP-1: CGGGAAAGATGAGGAAGAAGGG, producing a 450 bp PCR product. The MGP null gene mutation was detected using primers MGP-E4: TGCCTGAAGTAGCGGTTGTA and NEO-1: TGAATGAACTGCAGGACGAGG, producing a 1 kb PCR product. PCR conditions were 94 °C for 2 minutes followed by 94 °C for 45 s, 57 °C for 40 s, and 72 °C for 60 s for 35 cycles.

### 3D Computed Tomography

Whole animals were scanned using a Skyscan 1176 *in vivo* micro-CT scanner (Bruker microCT, Kontich, Belgium). All scans were taken at 50 kV, 500 µA with 0.5 mm aluminium filter, and 0.5 ° rotation step. Individual 2D cross-sectional images were reconstructed using Bruker NRecon software version 1.6.10.2. Voxel resolution was 18 µm. All reconstructed bones were set to the same orientation with the transverse plane perpendicular to the long axis of the bone using Bruker Dataviewer version 1.5.1.2. Reconstructed images were analysed using Bruker CTAn software version 1.16.4.1. Whole body scans from the C3 vertebra to the second caudal vertebra were used and reconstructed with a dynamic range of 0–0.08. Within CTAn, thresholds to determine body composition of fat, soft tissue, and bone were set at 17–24 (BAT 17–20; only measured within the interscapular region), 25–50, and 51–255 respectively. For the lungs, the chest cavity was reconstructed from a region within the trachea to just below the lower extremity of the lungs with a dynamic range of 0–0.025. Within CTAn, a volume of interest was produced by manually selecting around the area of the lung tissue. Within this new volume, the airways were selected using a threshold of 0–19, and lung tissue selected using a threshold of 20–60. The uppermost limit of the analysed volume was set as the dividing point of the bronchi. For the vertebra, the vertebral body of L3 was analysed. Height and width measurements were taken at the rearward end of the vertebra. For the femur, a section 1.76 mm in height and 0.7 mm behind the growth plate at the distal end was analysed for trabecular and cortical parameters, and the midshaft assessed for cortical parameters. For the tibia, a section 1.76 mm in height and 0.53 mm behind the growth plate at the proximal end was analysed for trabecular and cortical parameters.

The trabecular parameters measured were BvTv (volume of bone within a measured total volume), BsBv (surface to volume ratio of trabecular bone), trabecular thickness, number of trabeculae per mm, trabecular spacing (distance between trabeculae), structural model index (SMI, measure of surface convexity where a lower SMI is indicative of a more connected, plate-like trabeculae), trabecular pattern factor (indicator of connectivity of trabeculae where values closer to zero, both positive or negative, represent a more connected structure), and connectivity density.

### Sample Collection

#### Mice

Following the CT scan (detailed above), the thoracic aorta was removed, washed and flushed of blood in phosphate buffered saline (PBS) then immediately placed in Trizol Reagent (Invitrogen). The left femur was removed, cleaned of soft tissues, and cut in half. The marrow was flushed out of each half using PBS through a fine gauge needle (25 G). The halves were then cut into smaller pieces, approximately 2 mm in length, then immediately placed in Trizol Reagent.

### RNA Extraction and cDNA Production

Bone pieces were freeze/thawed twice whilst in Trizol Reagent, then shaken vigorously for 20 seconds every 2 minutes over a period of 10 minutes. RNA was extracted from samples in Trizol Reagent (Invitrogen) according to manufacturer’s instructions. The concentration and purity of RNA was determined by optical densities at 230 nm, 260 nm and 280 nm using a NanoDrop Spectrophotometer (Labtech, Uckfield, UK), and cDNA produced from RNA (500 ng for femur, 200 ng for aorta) using a Taqman reverse transcription kit with random hexamers (Applied Biosystems, Warrington, UK) following manufacturer’s instructions.

### Quantitative PCR

Relative quantification of gene expression was performed with an ABI Prism 7500 detection system (Applied Biosystems). The 20 μl reaction mixture was prepared, containing 1 μl of complementary DNA, 10 μl of GoTaq qPCR Master Mix (Promega, Southampton, UK), and 250 nM of each primer. Thermal cycler conditions consisted of an initial activation step at 95 °C for 10 min, followed by a 2-step PCR program of 95 °C for 15 s and 60 °C for 60 s for 40 cycles. A dissociation curve was obtained for each run. The 2−ΔΔCt method was employed for relative quantification of gene expression compared to the male C/C group, and the data were normalized to beta-actin, which was unaltered in its expression level between samples from the same site. The mouse primers used for qPCR were: *Mgp* forward, TCAACAGGAGAAATGCCAACAC; reverse, CGGTTGTAGGCAGCGTTGT; *Osteocalcin* forward, CTGACCTCACAGATGCCAAGC; reverse, TGGTCTGATAGCTCGTCACAAG; *Gas6* forward, TGCTGGCTTCCGAGTCTTC; reverse, CGGGGTCGTTCTCGAACAC; *Periostin* forward, CCTGCCCTTATATGCTCTGCT; reverse, AAACATGGTCAATAGGCATCACT; *Ggcx* forward, GTTGCTCCCGCCTCAGATAAA; reverse, TAAGCAGGGTCACGACACTCT; *Vkor* forward, GCTGGCTTAGCCCTCTCAC; reverse, CTGTCCGCTCCTAGCATGT; *Bmp-1* forward, TTGTACGCGAGAACATACAGC; reverse, CTGAGTCGGGTCCTTTGGC; *Bmp-2* forward, GTGCTTCTTAGACGGACTGC; reverse, TCAAATTCGCTGAGGACGTC; *Bmp-4* forward, TGTGAGGAGTTTCCATCACGA; reverse, CAGGAACCATTTCTGCTGGGG; *Sclerostin* forward, TCCTCCTGAGAACAACCAGAC; reverse TGTCAGGAAGCGGGTGTAGTG; *Leptin* forward, GAAAATGTGCTGGAGACCCC; reverse, GGTGAAGCCCAGGAATGAAG; *Leptin Receptor* forward, TGTGGTTTTGTTACACTGGGA; reverse, GGCATTGTTTGGGGCTCC; *Estradiol* forward, TCGGACAAACGAATTATGCGT; reverse, TTCCATGTCGTCCAAGCTCC; *Estradiol Receptor alpha* forward, TACTGTGCCGTGTGCAATGA; reverse, CTTTCCGTATGCCGCCTTTC; *Estradiol Receptor beta* forward, AGTGCGTGGAAGGGATTCTG; reverse, TCAGCTTCCGGCTACTCTCT; *Angiotensin Converting Enzyme (ACE)* forward, TATGACCGGACAGCCCAAGT; reverse, CCGGGTGCCATATTTCAGGG; *Angiotensinogen* forward, TGTCTAGGTTGGCGCTGAAG; reverse, GATGTATACGCGGTCCCCAG; *Vegf*_*120*_ forward, TGGCTTGTCACATTTTTCTGG; *Vegf*_164_ forward, CAAGGCTCACAGTGATTTTCTGG; *Vegf*_188_ forward, AACAAGGCTCACAGTGAACGCT; *Vegf*_*pan*_ reverse, GCCAGCACATAGGAGAGATGAGC; *Vegf*_*total*_ forward, ATCTTCAAGCCGTCCTGTGTG; reverse, GCATTCACATCTGCTGTGCTG; *Gapdh* forward, AGGTCGGTGTGAACGGATTTG; reverse, TGTAGACCATGTAGTTGAGGTCA; *Beta*-actin forward, AGCCATGTACGTAGCCATCCA; reverse, TCTCCGGAGTCCATCACAATG.

### Statistics

All data for all diet groups was observed to be normally distributed using the Shapiro-Wilks test. The effect of the gene knockout and sex was determined by two-way ANOVA followed by Bonferroni post hoc test using PASW version 21 (SPSS UK, Woking, Surrey, United Kingdom). Data are presented as mean ± Standard Deviation unless otherwise stated; significance was determined with a *P* value of 0.05 or lower. For variables correlated with size, we adjusted for differences using multiple regression for the variable of interest versus body mass and length and comparing residuals^37^. Relationships between variables were tested using linear regression analysis (univariant and multivariant) where a *P* value < 0.05 was considered statistically significant.

### Data Availability

The datasets generated during and/or analysed during the current study are available from the corresponding author on reasonable request.

## Electronic supplementary material

Supplementary Information
