# Supplementary material for: The influence of a high fat diet on bone and soft tissue formation in Matrix Gla Protein knockout mice
Source: Sci Rep. 2018 Feb 26;8:3635. doi: 10.1038/s41598-018-21650-0 (PMC5827663; doi:10.1038/s41598-018-21650-0)
Supplement: Supplementary file 1 — Supplementary Information [file 41598_2018_21650_MOESM1_ESM.doc]

**The influence of a high fat diet on bone and soft tissue formation in Matrix Gla Protein knockout mice**

S.A. Lanham, F.R. Cagampang and R.O.C. Oreffo.

**Supplemental Material**

Vascular Calcification


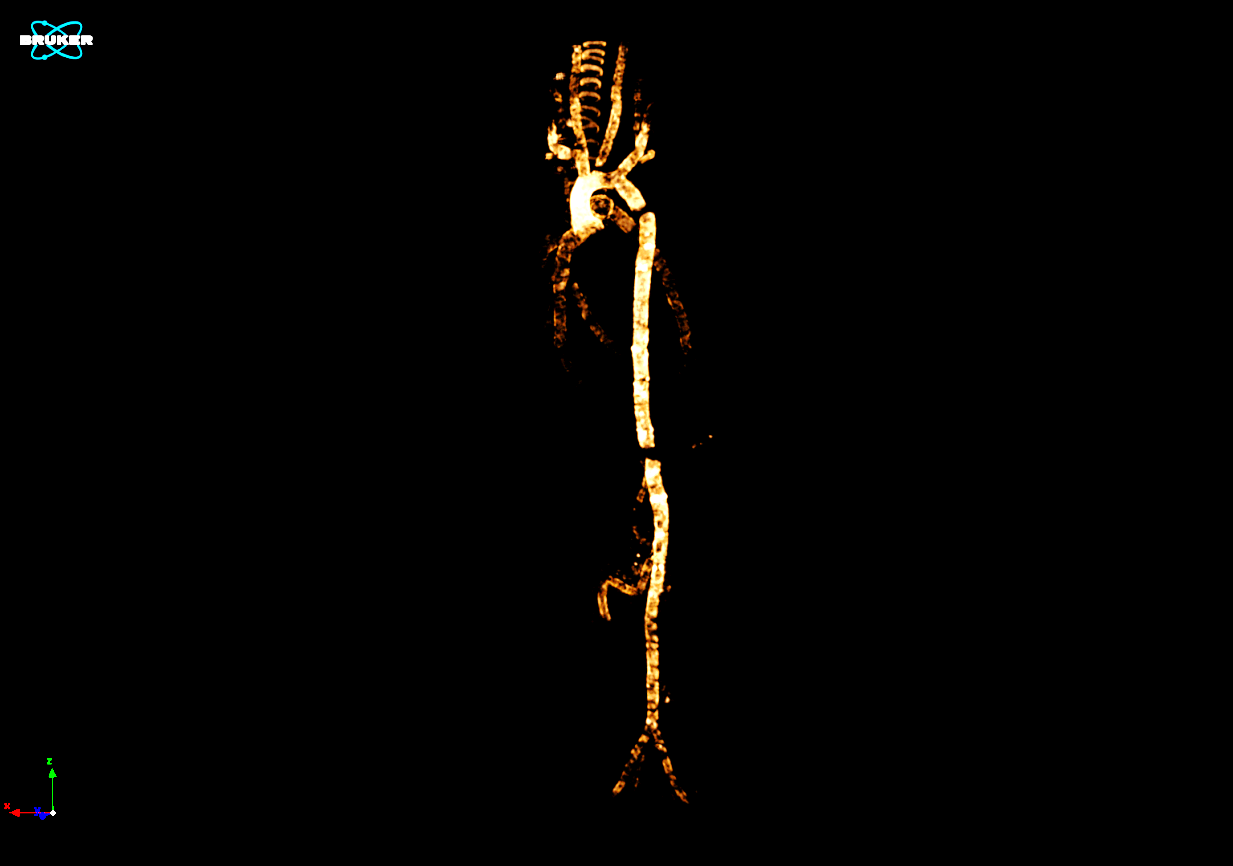

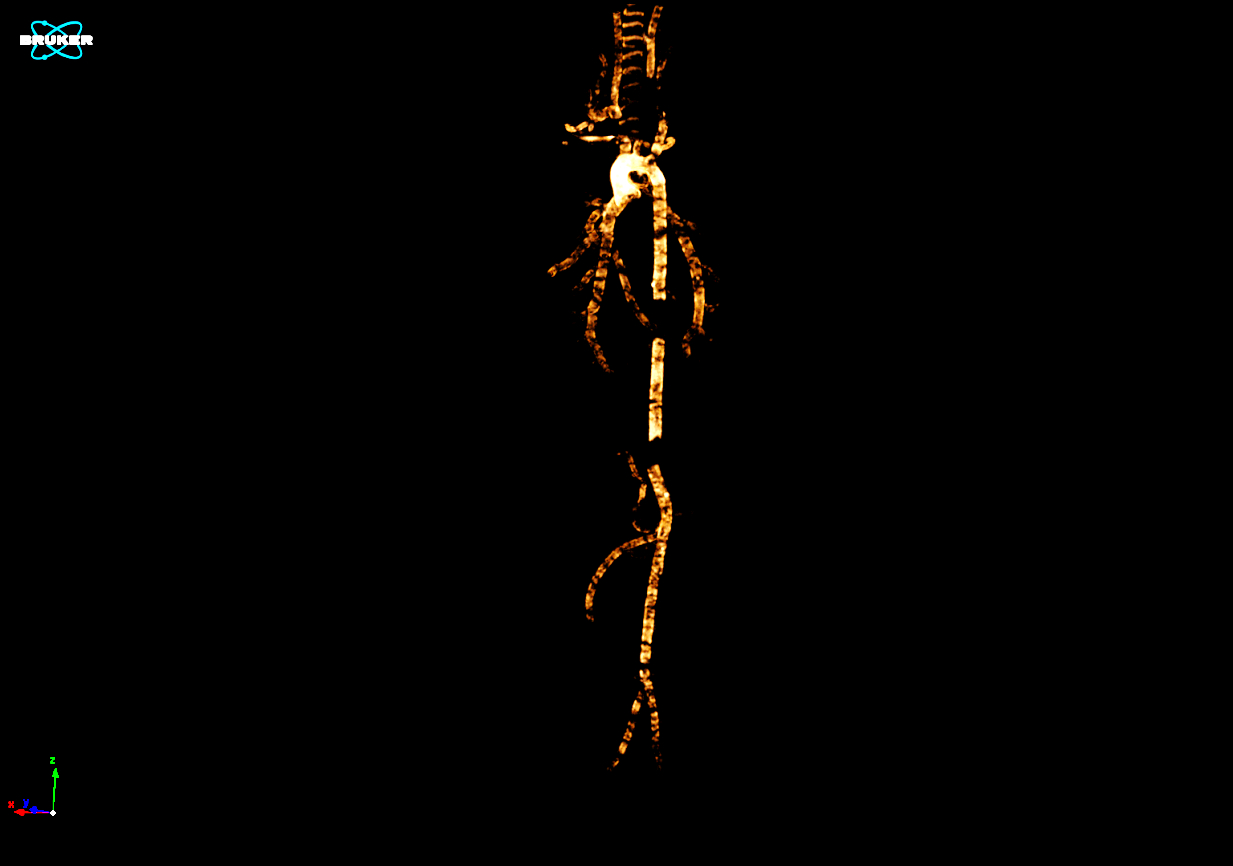


KO C

KO HF

Representative images of vascular calcification from CT scans. Only KO animals showed vascular calcification. Control fed animals are shown on the left panel, and high fat fed animals on the right panel.

Lung Tissue

WT C

WT HF

KO C

KO HF


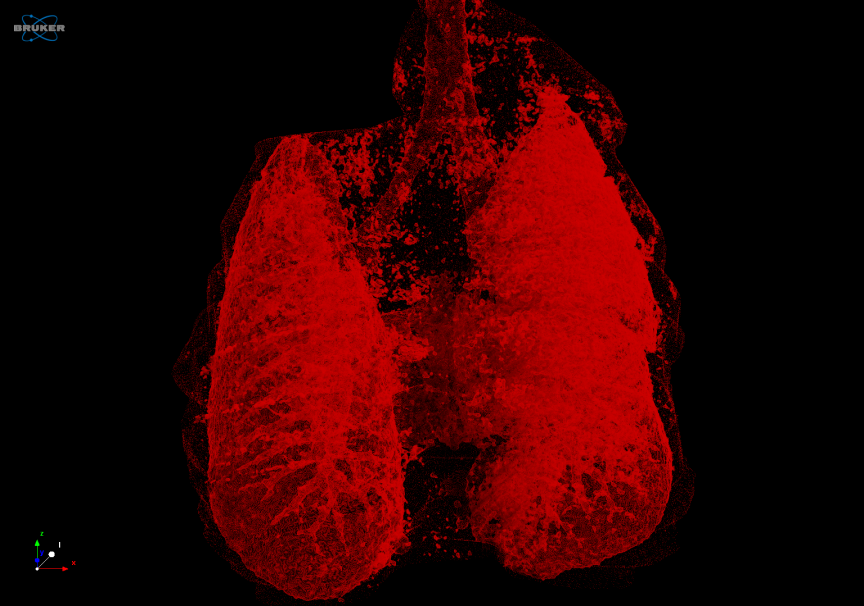

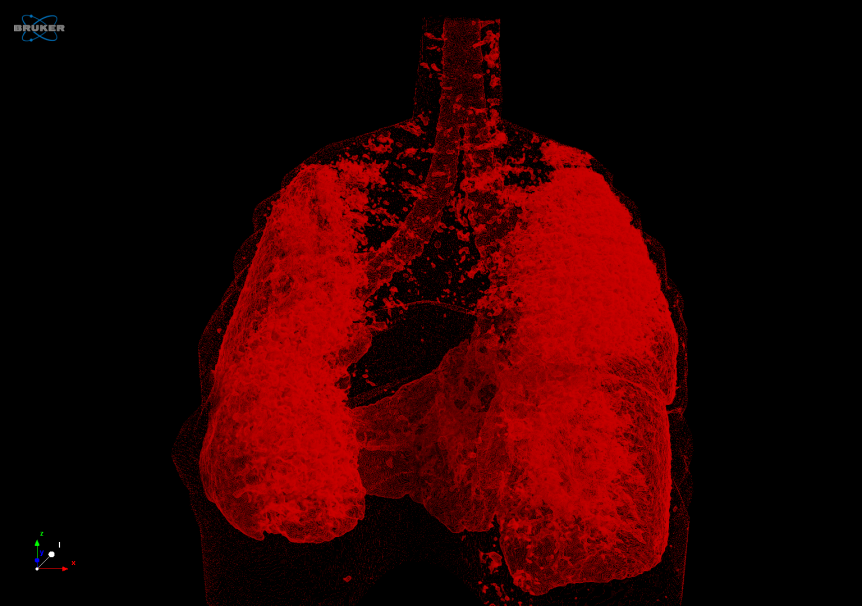

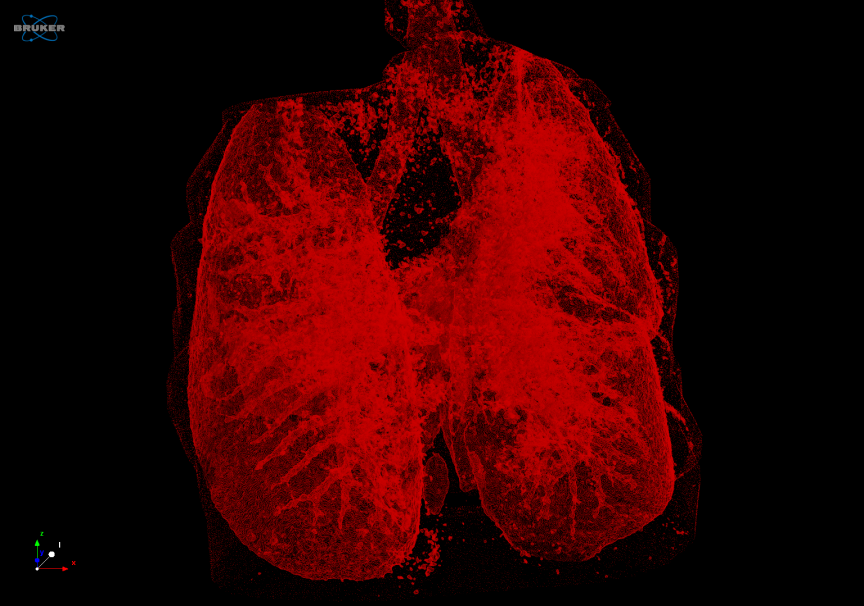

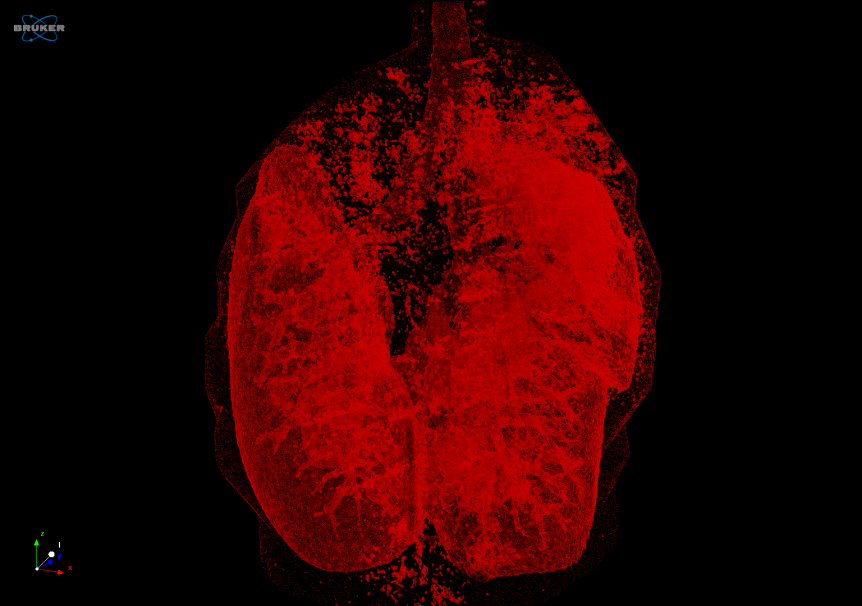


Representative images of lung tissue from CT scans. Scans from WT animals are shown in the left column and KO animals are in the right column. Control fed animals are shown on the top row, and high fat fed animals on the bottom row. The yellow box has the same dimensions in all images and is used as a size reference between scans.

Lung Airways

WT C


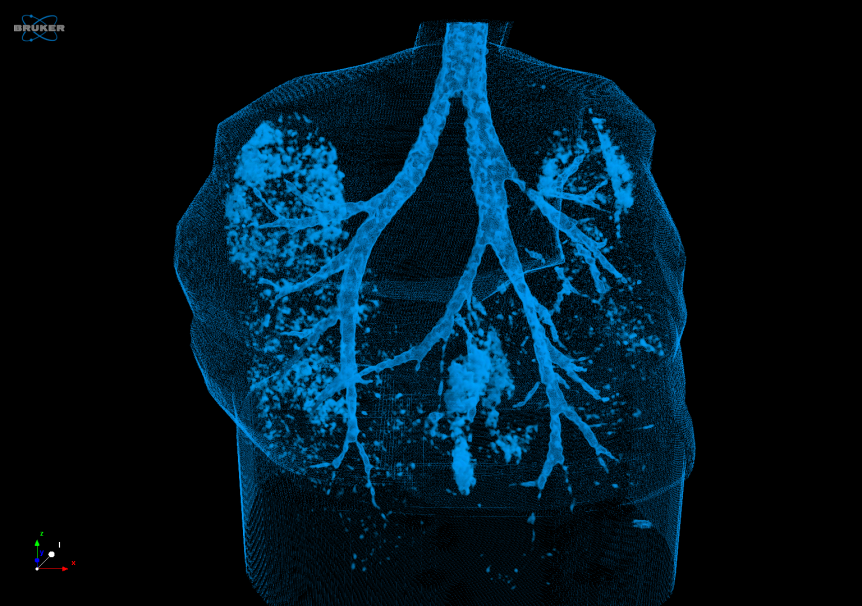


WT HF


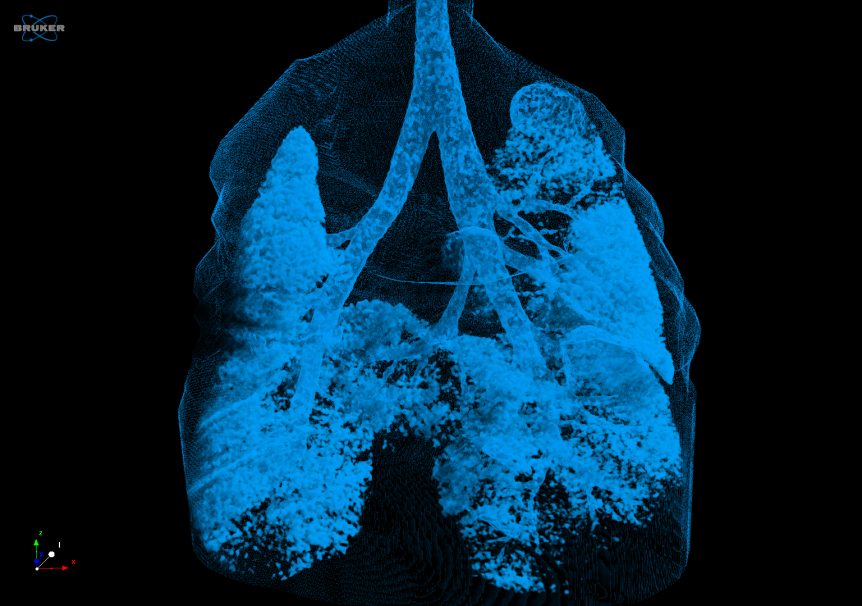


KO C


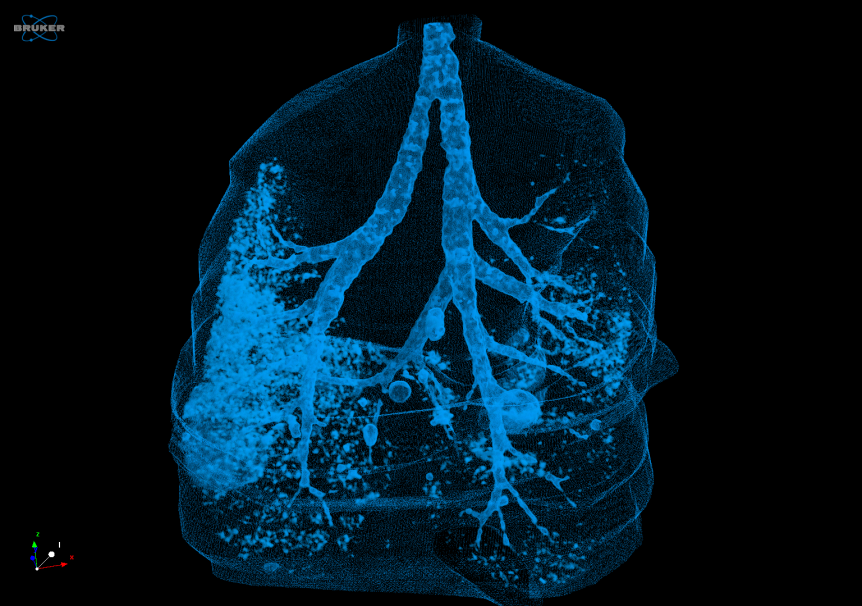


KO HF


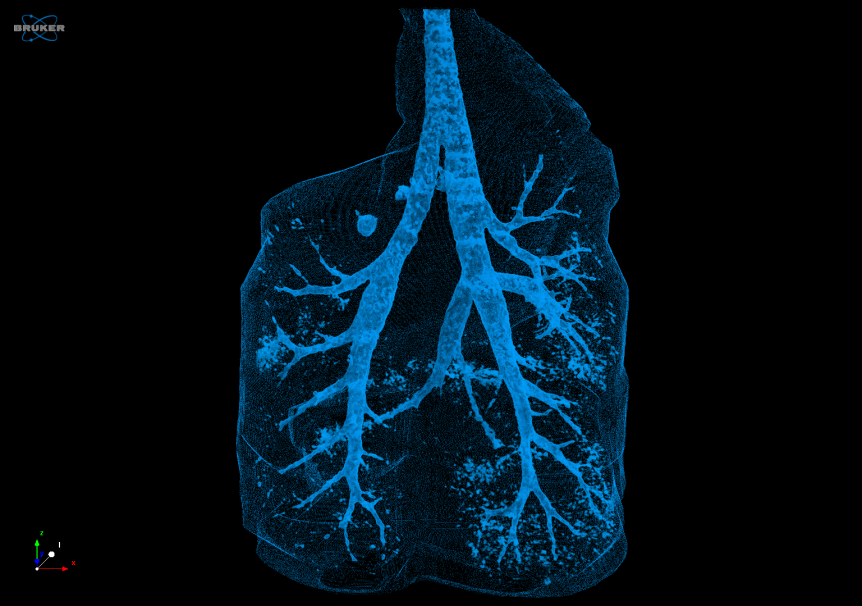


Representative images of lung airways from CT scans. Scans from WT animals are shown in the left column and KO animals are in the right column. Control fed animals are shown on the top row, and high fat fed animals on the bottom row. The yellow box has the same dimensions in all images and is used as a size reference between scans.
